# Evidence of batch effects masking treatment effect in GAW20 methylation data

**DOI:** 10.1186/s12919-018-0129-6

**Published:** 2018-09-17

**Authors:** Angelo J. Canty, Andrew D. Paterson

**Affiliations:** 1 Department of Mathematics and Statistics, McMaster University, 1280 Main St. W., Hamilton, ON L8S 4K1 Canada; 2 Genetics and Genome Biology Program, The Hospital for Sick Children Research Institute, 686 Bay St., Toronto, ON M5G 0A4 Canada; 3 Dalla Lana School of Public Health, University of Toronto, Toronto, ON M5T 3M7 Canada

## Abstract

Using the real data set from GAW20, we examined changes in the distribution of DNA methylation before and after treatment. Paired analysis of differences in both mean and variance had grossly inflated type 1 error, suggesting either a very large number of changes across the entire epigenome or major non-biological issues, such as batch effects. Separate analysis of Infinium I and II probes indicated differences in the paired *t*-test statistics between these two types of probes. Examination of combined principal components showed that the first and fourth principal components discriminate between the before and after treatment measurements, further evidencing the presence of batch effects that make any conclusions about treatment effect suspect.

## Background

Treatment of CD4+ T cells with fenofibrate results in differences in gene expression and interferon γ protein levels [1], suggesting some of its actions may be mediated by effects on DNA methylation. For the GAW20 data, the Illumina Human Methylation 450 K BeadChip was used to measure methylation in CD4+ T cells before and after 3 weeks of treatment with 160 mg oral fenofibrate. This chip uses two different probe chemistries (Infinium Type I and Infinium Type II) to assess methylation [2]. The two probe types have differing dynamic ranges and target different genomic features [3]. The supplied data gave the normalized methylation proportion (β values) at each of 463,995 cytosine-phosphate-guanine (CpG) sites. Previously, the Genetics of Lipid Lowering Drugs and Diet Network (GOLDN) study examined the association of change in lipids, before and after treatment, with change in DNA methylation [4]. No genome-wide significant associations were observed. In that analysis, methylation measures before and after treatment were normalized (separately at each time point, stratified for Type I and Type II probes) and adjusted for differences in cell composition using the first four principal components. In our analysis, we examined the differences in methylation, and also adjusted for principal components and change in triglyceride levels to examine changes in the distribution of methylation before and after treatment.

## Methods

Methylation measures were available at both time points for 446 individuals across 140 pedigrees. To avoid complications resulting from relatedness, we selected one individual at random from each of these pedigrees, and so used a sample of *n* = 140 for our analyses. Because the original β values are non-normal we used a logit transformation to get M-values as suggested by Du et al. [5]. We omitted 668 probes that gave infinite means on the logit scale, leaving 463,327 sites for analysis.

We were interested in looking for evidence of differences in both the mean and variability of the methylation values. For differences in the mean, we used a simple paired *t* test at each site as our primary analysis. To examine differences in the variability we used the Pitman-Morgan test [6]. Theory shows that the covariance between the sum and difference of two random variables is equal to the difference in their marginal variances. This result implies that to test for the equality of variance in a paired setting, we need to test the hypothesis that the correlation between the sum of the pre- and post-treatment methylation values and their difference is equal to zero. We used the usual *t* test of zero correlation between normal random variables for this. Both tests described above rely on the assumption of normality of the underlying M-values. As a sensitivity analysis, we also replaced the two *t* tests with non-parametric tests: the Wilcoxon signed rank test in place of the paired *t* test, and the Spearman’s rank correlation test in place of the *t* test for correlation.

To examine the impact of principal components and the change in triglycerides we also recast both the paired *t* test and the correlation test in terms of a standard linear model with the difference in methylation being the response variable. The usual paired *t* test is equivalent to a test of 0 intercept in such a model, and the test of correlation is equivalent to a test of a zero slope for the sum when it is included as a covariate in the model.

## Results

### Difference in mean methylation

**Fig. 1:**
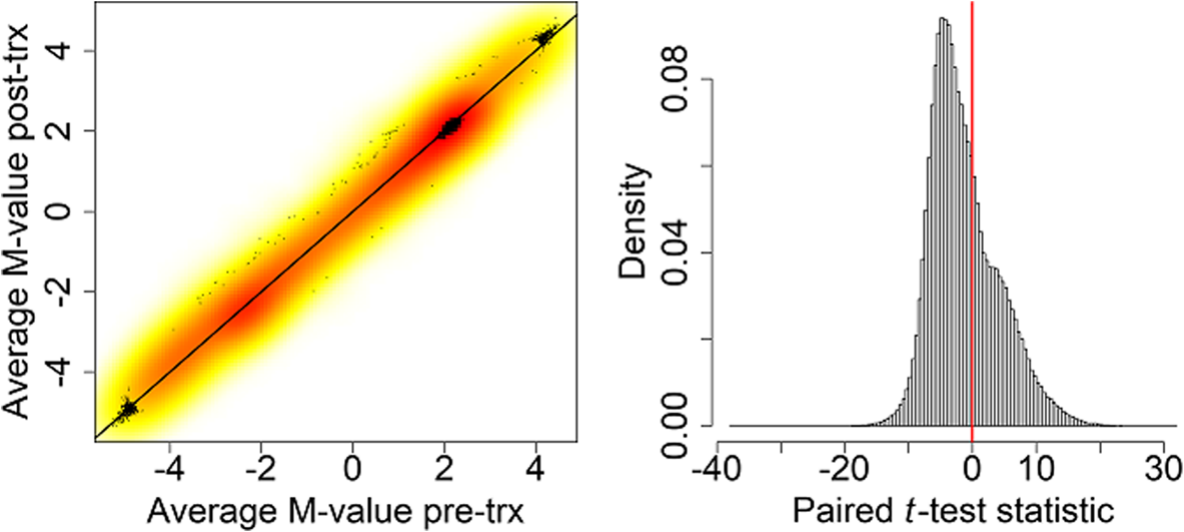
Density-smoothed scatterplot of the average M-values pre- and post-treatment with the line of equality (*left panel*) and histogram of the paired *t*-test statistic for each of the 463,327 probes with a vertical line at 0 (*right panel*). trx, Treatment

Figure 1 shows a comparison of the methylation M-values before and after treatment, as well as the distribution of the paired *t*-test statistic for each of the 463,327 probes analyzed.

**Fig. 2:**
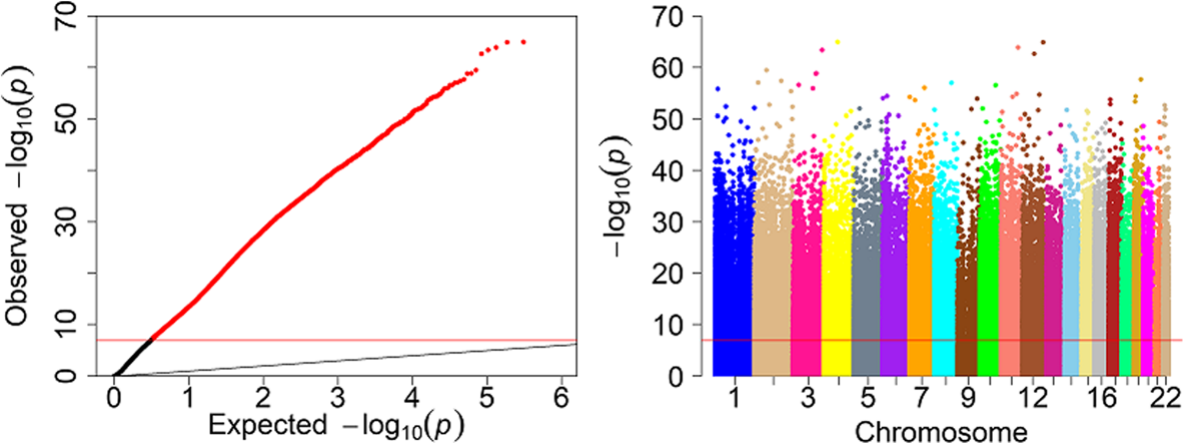
Quantile-quantile plot of the paired *t* test *p* values on the −log_10_ scale *(left)* and the Manhattan plot of the −log_10_ (*p* values) against genomic position with chromosomes in different colors *(right)*. The horizontal red line in each plot is the Bonferroni-corrected significance level. The thin black diagonal line in left figure is x = y

Methylation decreased during the course of the treatment for 65.4% of the probes. There also seems to be a shoulder in the histogram of the paired *t* test statistic, suggesting a possible mixture of two distributions. Figure 2 shows a quantile-quantile (−log_10_ scale) of the resulting *p* values and a Manhattan plot. There is very clear inflation of Type 1 error (λ = 36.18). In fact, 32.3% of probes had a significant *p* value after Bonferroni correction as shown by the red horizontal lines on the panels of Fig. 2.

We found a very similar distribution of *p* values when using the Wilcoxon test (results not shown) indicating that deviation from normality is not the cause of the excess of small *p* values. We conclude that there are real differences between the observed methylation signals pre- and post-treatment.

### Difference in variance of methylation

**Fig. 3:**
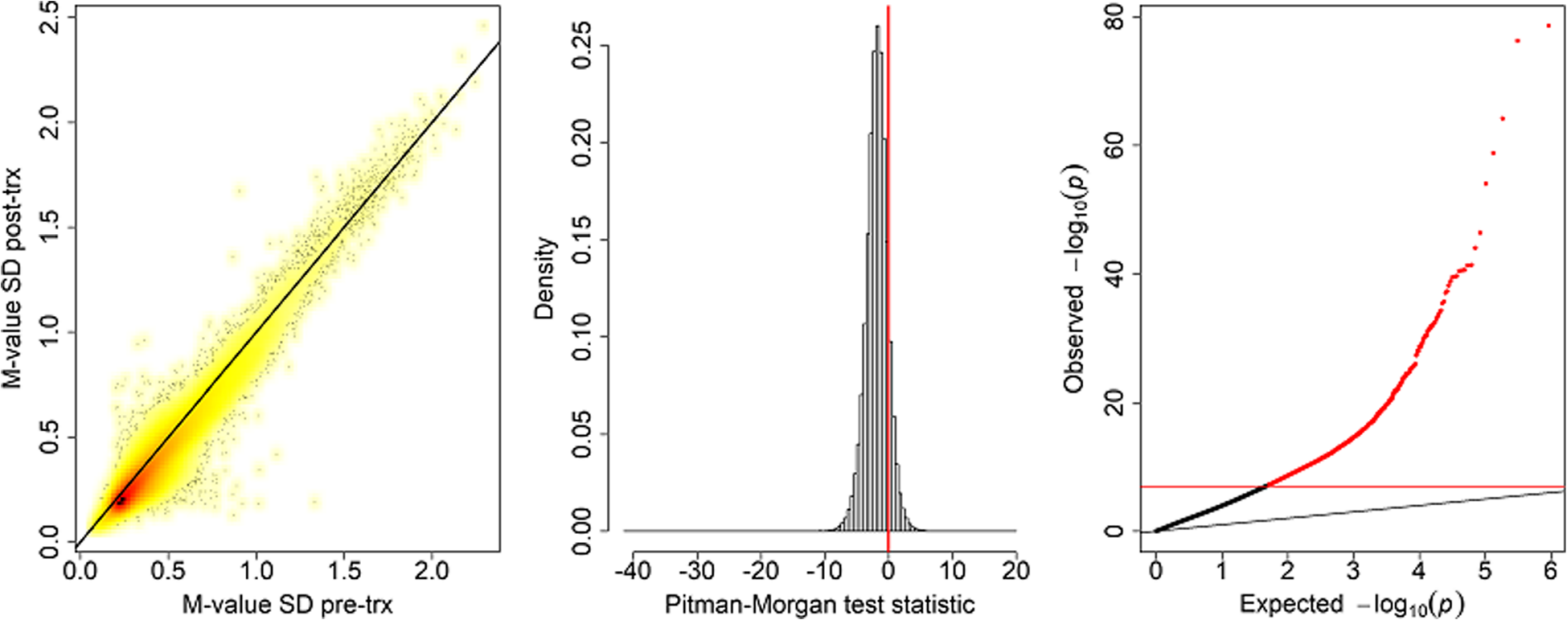
Density scatterplot of the standard deviations of the M-values pre- and post-treatment *(first panel)*, a histogram of the test statistic for equality of variances *(second panel)*, and a quantile-quantile plot of the −log_10_ (*p* values) *(third panel)* with the Bonferroni-corrected significance level as a horizontal red line. The black diagonal line in the first and third panels is x = y

Figure 3 shows plots of the standard deviation of the logit methylation at the two time points and a histogram of the test statistic which shows that for most probes the variability of methylation signal decreased after treatment. The quantile-quantile plot of Pitman-Morgan test *p* values in the third panel of Fig. 3 again shows an excess of small *p* values (λ = 7.61). We found 9982 probes (2.2%) that showed significant differences in standard deviation (SD). Almost all (9807) of these significant probes had higher SD pre-treatment. There was no material change to the results when a non-parametric test was used in place of the *t* test (results not shown).

### Probe type analysis

**Fig. 4:**
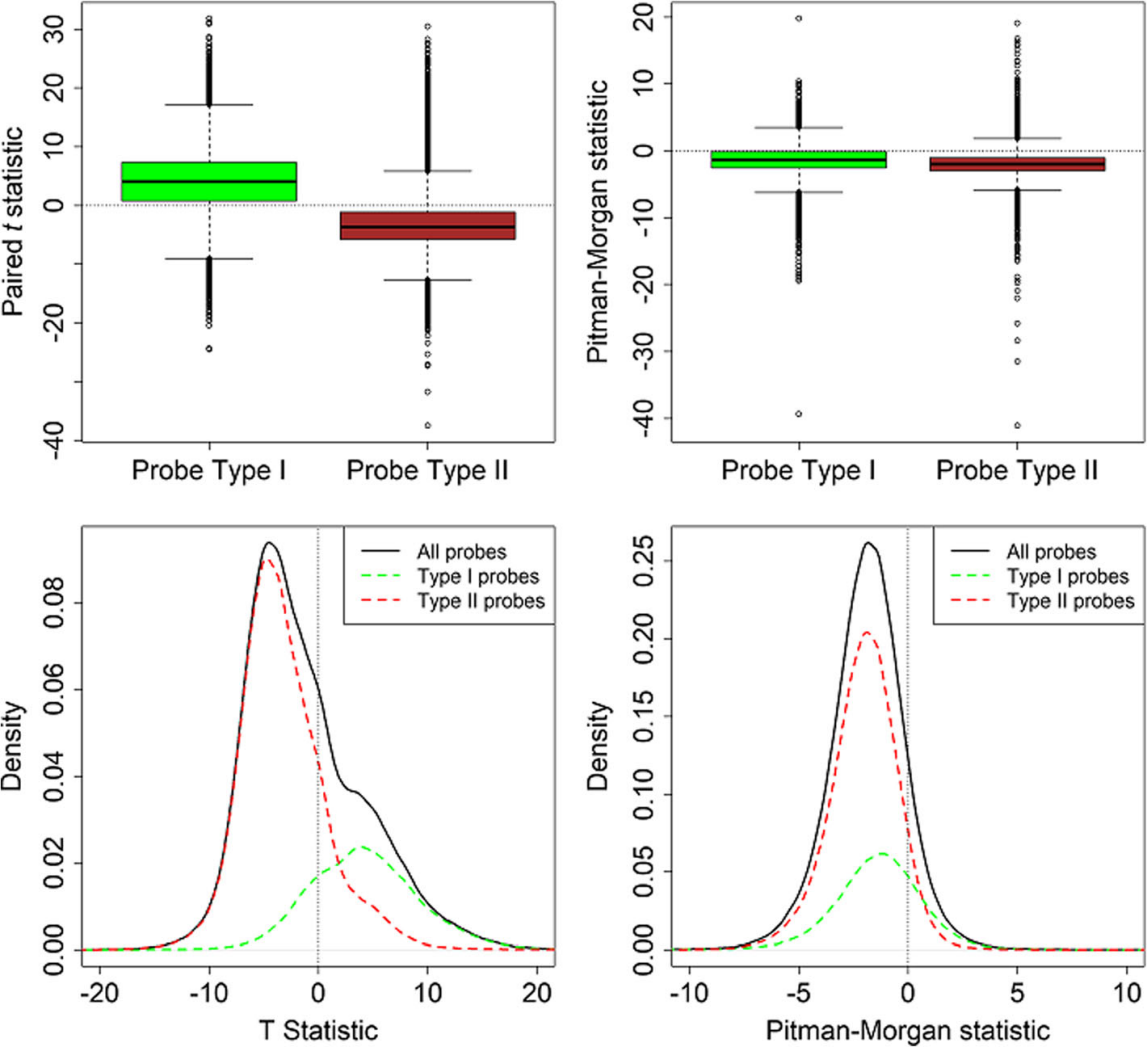
Boxplots of the two test statistics used stratified by probe type. *Top row:* Boxplots of the paired *t (left panel)* and Pitman-Morgan *(right panel)* test statistics stratified by Infinium probe type. *Bottom row:* Scaled kernel density estimates of the paired *t (left panel)* and Pitman-Morgan statistics *(right panel)* for all probes and stratified by probe type

There are two probe types on the Illumina Human Methylation 450 K BeadChip. We examined the relationship between probe type and methylation difference. We analyzed 128,310 Type I and 335,017 Type II probes. Figure 4 shows boxplots of the two test statistics used, stratified by probe type. In the left panel of the plot we see a marked difference in distribution for the two probe types. Whereas most Type I probes showed an increase in mean after treatment, most Type II probes showed a decrease. This explains the shoulder seen in the overall histogram in the right panel of Fig. 1 as shown by the kernel density plots in the bottom left panel of Fig. 4. For the paired test of equality of variance in the right panels of Fig. 4 we do not see any major differences by probe type.

### Principal component analysis

**Fig. 5:**
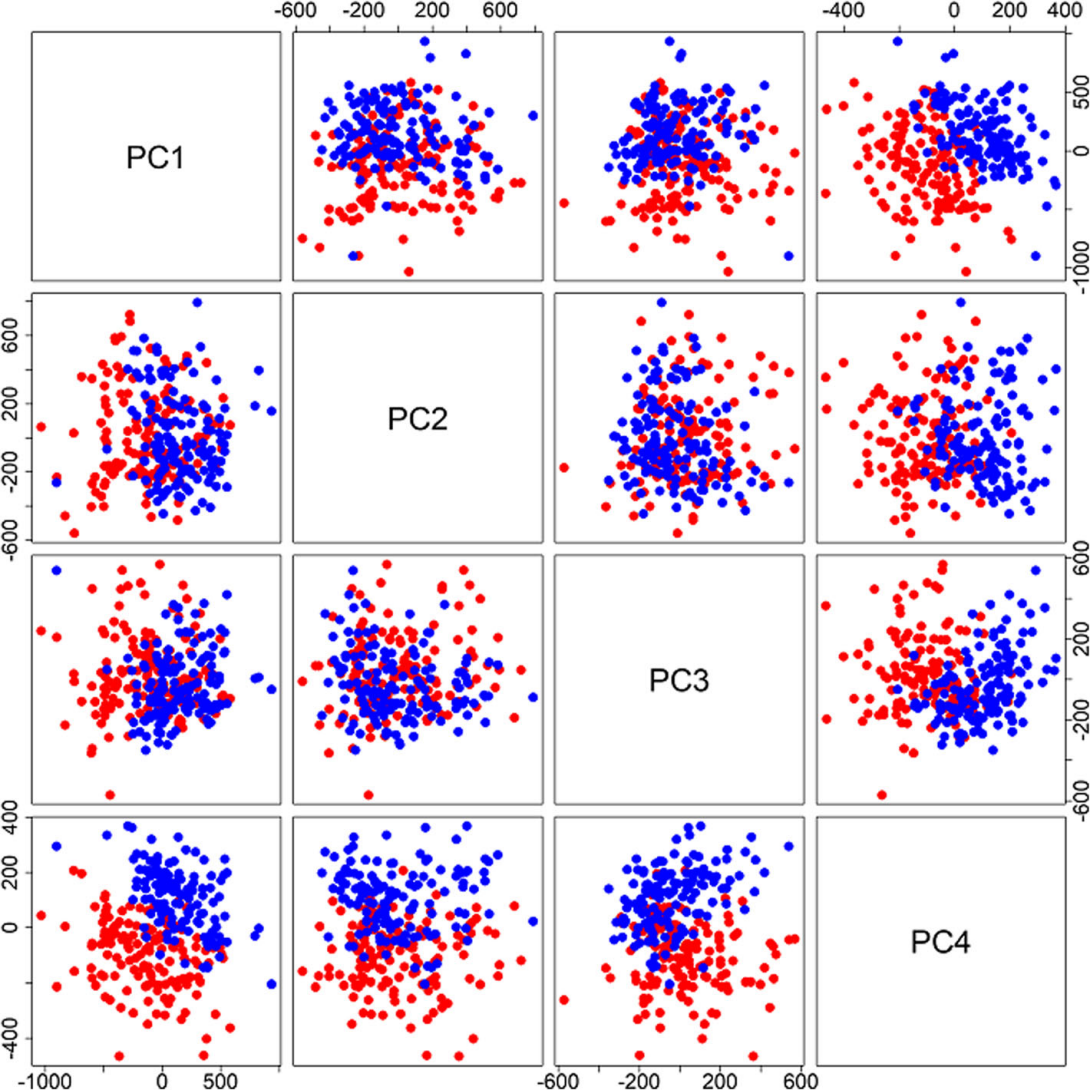
Pairwise scatterplots of the PCs calculated using the logit of the methylation beta values for the two visits. The values of the PCs for the pre-treatment observations are shown in red, and those for the post-treatment observations are shown in blue

Das et al. [4] attempted to correct for differences in T-cell purity and batch effects by adjusting the methylation values for the first 4 principal components (PCs). To generate PCs we considered both sets of methylation M-values jointly. We combined the values from the 2 arrays per person into a single 280 × 463,327 matrix, and calculated the joint PCs from this data set. Figure 5 shows pairwise scatterplots of the first 4 joint PCs with different colors for the 2 time points. The first and fourth PCs together almost completely separate the pre- and post-treatment measures.

### Controlling for triglyceride differences

**Fig. 6:**
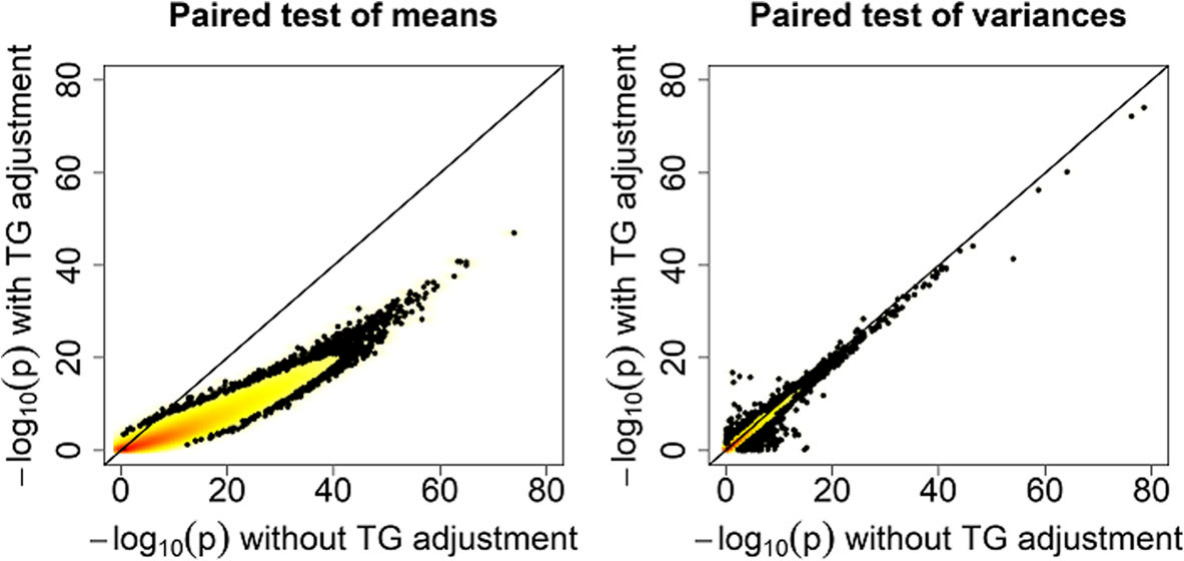
Comparison of −log_10_ (*p* values) for the paired tests of means *(left panel)* and variances *(right panel)* before and after adjustment for the difference in log triglycerides (TG)

Because the main expected effect of fenofibrate treatment is to lower triglyceride levels, we decided to consider the tests adjusted for the difference in log-transformed triglycerides. If the effects we are seeing are caused by an effect of fenofibrate, we would expect this to remove any such effects. Figure 6 compares the −log_10_ (*p* values) before and after adjustment for the difference in log-triglycerides. Figure 6 shows that the *p* values after adjustment tend to be less extreme for the test of means, and marginally so for the test of variances. Despite this attenuation of the small *p* values problem, there still remain 26,371 (5.7%) significant probes for differences in mean and 7856 (1.7%) significant probes for differences in variance. These differences are distributed across every chromosome, as we saw in the data before adjustment. Further adjustment of the tests for the first 4 PCs of the pre-treatment M-values, as well as those for the post-treatment M-values, had minimal impact on the distribution of genome-wide *p* values (results not shown).

## Discussion

We see a very large number of significant differences in mean and variance of the methylation distribution before and after fenofibrate treatment. The distribution of the paired *t* test statistics is asymmetric and varies by probe type. PCs almost completely separate the data from the two visits. We believe that the large differences in the mean and variance of the methylation values seen across the epigenome are unlikely to be caused by the treatment; rather, they suggest systematic batch effects between the processing of the samples from the two visits. If the observed differences really were caused by the treatment then we would expect them to be highly correlated with the major treatment effect, namely difference in the triglyceride level. Adjustment of the tests for differences in log-triglycerides attenuates, but does not remove, the issue of an excess of very small *p* values across the genome. Other authors, such as Bock [7], have also commented on the presence of major batch effects when arrays are processed at different times. Our understanding is that the pre-treatment arrays were all processed and normalized first, and the post-treatment arrays were processed and normalized later. Joint normalization of the original data across both time points may have helped to correct for some of these systematic differences, but the data that would have allowed joint normalization was not available as part of the GAW20 data set used in this analysis.

## Conclusions

It is our view that the batch effects seen in the GAW20 methylation data make it impossible to draw any real conclusions regarding the differences in methylation or their association with other traits. These effects are likely to occur in any longitudinal analysis of methylation, and so care needs to be taken to minimize the effects by processing and normalizing all arrays together for any analysis that will look at changes over time.
